# Development of Retroviral Vectors for Tissue-Restricted Expression in Chicken Embryonic Gonads

**DOI:** 10.1371/journal.pone.0101811

**Published:** 2014-07-08

**Authors:** Luke S. Lambeth, Thomas Ohnesorg, David M. Cummins, Andrew H. Sinclair, Craig A. Smith

**Affiliations:** 1 Murdoch Childrens Research Institute, Royal Children's Hospital, Melbourne, VIC, Australia; 2 Department of Paediatrics, The University of Melbourne, Melbourne, VIC, Australia; 3 Poultry Cooperative Research Centre, Armidale, NSW, Australia; 4 CSIRO Animal, Food and Health Sciences, Australian Animal Health Laboratory, Geelong, VIC, Australia; University of Maryland School of Medicine, United States of America

## Abstract

The chicken embryo has long been a useful model organism for studying development, including sex determination and gonadal differentiation. However, manipulating gene expression specifically in the embryonic avian gonad has been difficult. The viral vector RCASBP can be readily used for embryo-wide transgene expression; however global mis-expression using this method can cause deleterious off-target effects and embryo-lethality. In an attempt to develop vectors for the over-expression of sequences in chicken embryonic urogenital tissues, the viral vector RCANBP was engineered to contain predicted promoter sequences of gonadal-expressed genes. Several promoters were analysed and it was found that although the *SF1* promoter produced a tissue-restricted expression pattern that was highest in the mesonephros and liver, it was also higher in the gonads compared to the rest of the body. The location of EGFP expression from the *SF1* promoter overlapped with several key gonad-expressed sex development genes; however expression was generally low-level and was not seen in all gonadal cells. To further validate this sequence the key testis determinant DMRT1 was over-expressed in female embryos, which due to insufficient levels had no effect on gonad development. The female gene aromatase was then over-expressed in male embryos, which disrupted the testis pathway as demonstrated by a reduction in AMH protein. Taken together, although these data showed that the *SF1* promoter can be used for functional studies *in ovo*, a stronger promoter sequence would likely be required for the functional analysis of gonad genes that require high-level expression.

## Introduction

One of the main strengths of using chickens and other avian species for studies in development is the ability to manipulate an embryo that develops outside the maternal body. Numerous techniques can be used to track and manipulate factors involved in embryonic development and differentiation. These include the transplantation of tissues, implantation of beads soaked with growth factors, and the introduction of recombinant DNA by electroporation and retroviruses [1].

There are three principal methods for gene transfer into the developing chicken embryo; transfection, viral infection and electroporation. *In ovo* electroporation has been widely described and allows for controlled gene expression both temporally and spatially. Various areas of the embryo can be specifically targeted by electroporating different tissues and stages of development, including the midbrain [2], the somites [3], the retina [4] the spinal cord [5], [6], and the forelimb mesoderm [7]. Although the targeting of intermediate mesoderm or primordial gonads by electroporation has been described, this method is generally not very well established. Electroporation of viral vectors has been used to achieve ectopic PITX2 expression in the gonads of developing chicken embryos [8], and more recently the developing left gonad was targeted for overexpression of DMRT1 [9]. Another approach to targeting gonadal expression has exploited the transfection of migrating primordial germ cells. Lipofection of recombinant Tol2 transposon and transposase plasmids into very early stage (14HH) chicken embryos, results in effective integration in primordial germ cells, which subsequently migrate to the developing gonads and deliver GFP reporter expression [10].

The most widely used retroviral system used in avian developmental studies is RCAS (Replication Competent ALV LTR with a Splice acceptor), which is a modified version of an avian Rous sarcoma virus [11]. A cDNA copy can be inserted downstream of the viral *env* gene which is transcribed by a promoter within one of the viral long terminal repeats (LTRs) and subsequently spliced. Infection with RCAS permits sustained mis-expression of inserted sequences following the stable integration of the viral DNA into the host genome. Infection at early developmental stages or of highly proliferative cell populations can result in large areas of the embryo being infected. Indeed, injection of an RCASBP (a modified RCAS with Bryan RSV Polymerase) encoding EGFP into the blastoderms of susceptible eggs results in embryo-wide EGFP expression, including the urogenital systems of both male and female embryos [12]. Despite the considerable potential for experimental analysis of candidate genes using this method, embryo-wide overexpression also could induce unwanted off-target effects. Many of the key factors involved in sex development are transcription factors, such as the male up-regluated genes DMRT1 and SOX9, and the female up-regulated gene FOXL2. Embryo-wide over-expression of these genes is expected to be lethal, and indeed we have found this to be the case when RCASBP was used to deliver DMRT1 or FOXL2 (CA Smith, unpublished data). In contrast, knockdown of DMRT1 in chicken embryos was achieved using a U6 promoter to express shRNAs from RCASBP, resulting in feminization of male gonads [13]. Although the shRNA was expressed throughout the embryo, the urogenital system restricted expression pattern of DMRT1 meant that this protein was suppressed in these tissues only, and no overt off-targeting effects were noted.

The viral vector RCANBP is derived from RCASBP, however it lacks the splice acceptor site downstream of the *env* gene. This vector thereby permits the introduction of exogenous internal promoter sequences to direct transgene expression instead of the viral LTR. Infection of RCANBP that contains EGFP under control of the human cytomegalovirus (CMV) promoter results in widespread EGFP expression in E8.5 embryos [14]. Although this expression is strong, it is in restricted regions including the retinal-pigmented epithelium, liver, and proliferating zones in developing bones. In contrast, EGFP expression from RCASBP/EGFP is generally more widespread throughout the embryo [14].

The manipulation of genes in embryonic chicken gonads is of interest to the field of sex determination and differentiation. In the chicken and all other birds, a ZZ male: ZW female sex chromosome system exists, the inheritance of which, determines sex. The exact molecular mechanisms leading to sexual differentiation however, are still not fully understood. Gonad sexual differentiation appears to be highly conserved and many of the important signaling factors involved in ovarian or testis development in mammals are also implicated in birds [15]. Therefore, gonad-specific expression of key sex development factors would help to further advance studies in chicken sex determination. To address this, we have identified and compared the ability of several gonad-expressed gene promoters to drive reporter expression in the embryonic urogenital system of chicken embryos.

## Results

### Promoter characterisation

To develop retroviral vectors for tissue-specific expression in chicken embryonic gonads, promoter regions of several genes that are expressed in embryonic gonads were characterised. These genes included: Wilm's tumor suppressor *(WT1)*, Steroidogenic factor 1 (*SF1*, *NR5A1*), Anti-Müllerian hormone *(AMH) and* aromatase *(CYP19A1)*. The DNA sequences of the regions located directly upstream of each open reading frame were obtained from the UCSC Genome Browser from the Chicken May 2006 (WUGSC 2.1/galGal3) Assembly.

**Figure 1:**
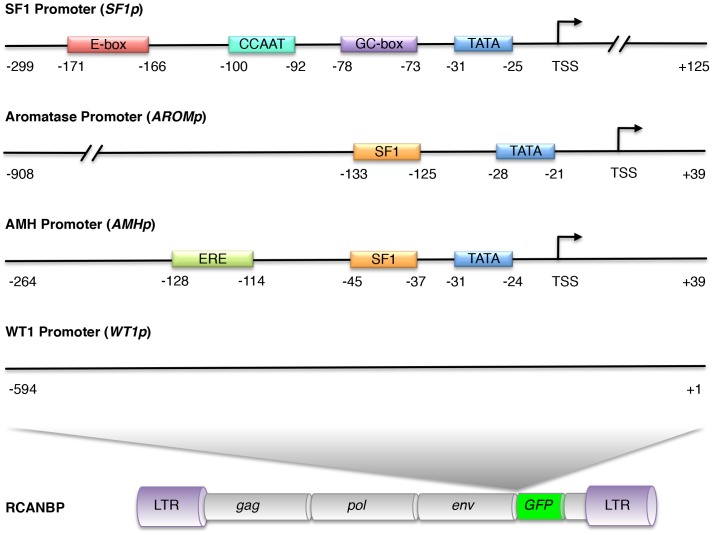
Schematic representation of putative gonad promoter sequences. All numbers shown are relative to the transcriptional start site (TSS) for each putative promoter sequence. The *SF1p* contains several promoter elements that have been described previously [16]. Both *aromatase* and *AMH* promoters contain TATA boxes and consensus SF1 binding sites. The *AMH* promoter also contains an estrogen responsive element (ERE). The *WT1* promoter is TATA-less and no other binding elements were identified. All promoter sequences were cloned into the RCANBP viral vector directly upstream of the EGFP open reading frame.

For each putative promoter region, the sequence was analysed for the presence of potential regulatory elements and transcription factor binding sites (Figure 1). An analysis of the putative chicken *SF1* promoter was previously described, which reported the identification of several promoter elements and its activity *in vitro* [16]. In the current study, a 424 nt region directly upstream of the chicken *SF1* coding sequence that included 125 nt of sequence downstream of the predicted TSS (accession AB018710) was cloned and verified. Binding elements including a TATA-box, GC-box, CCAAT-box and an E-box were identified as described previously [16]. For the aromatase promoter (*AROMp*), a 947 nt region upstream of the aromatase coding sequence was cloned that included 39 nt of sequence downstream of the TSS in ovary [17]. Analysis of this sequence revealed a TATA box from −28 to −21 and an SF1 consensus-binding site from −133 to −125. The SF1 binding site was the same sequence as the SFRE consensus sequence [18] and is very similar to those found in the mouse 3β-Hydroxysteroid dehydrogenase 1 (HSD1) and Cyp17 promoters [19]. The chicken *AMH* promoter was previously characterised and analysed for the presence of potential SOX9 binding sites [20]. In this study, a 303 nt region directly upstream of the coding sequence was cloned that included 45 nt of sequence downstream of the TSS. Consistent with the study by Oreal et al., features within the *AMHp* region included a TATA box and an estrogen responsive element (ERE), as well as a consensus SF1 binding site (5′-TCAAGGCCA-3′). To isolate a putative chicken *WT1* promoter sequence (*WT1p*), a region of 594 nt directly upstream of the predicted *WT1* coding sequence was cloned. Like the human *WT1* promoter [21], this sequence did not have a TATA-box or a CCAAT-box, and we were not able to identify any typical gonad transcription factor consensus binding sites. In addition to the chicken promoters, the mouse *SF1* promoter was also used to drive EGFP expression from RCANBP. This sequence was previously described and tested in mice, where it produces strong and specific expression in mouse gonads [22]. To act as a positive control for EGFP expression from RCANBP using an internal promoter, the well characterised and widely used Simian virus 40 (*SV40*) promoter was also included.

### Promoter validation in ovo

**Figure 2:**
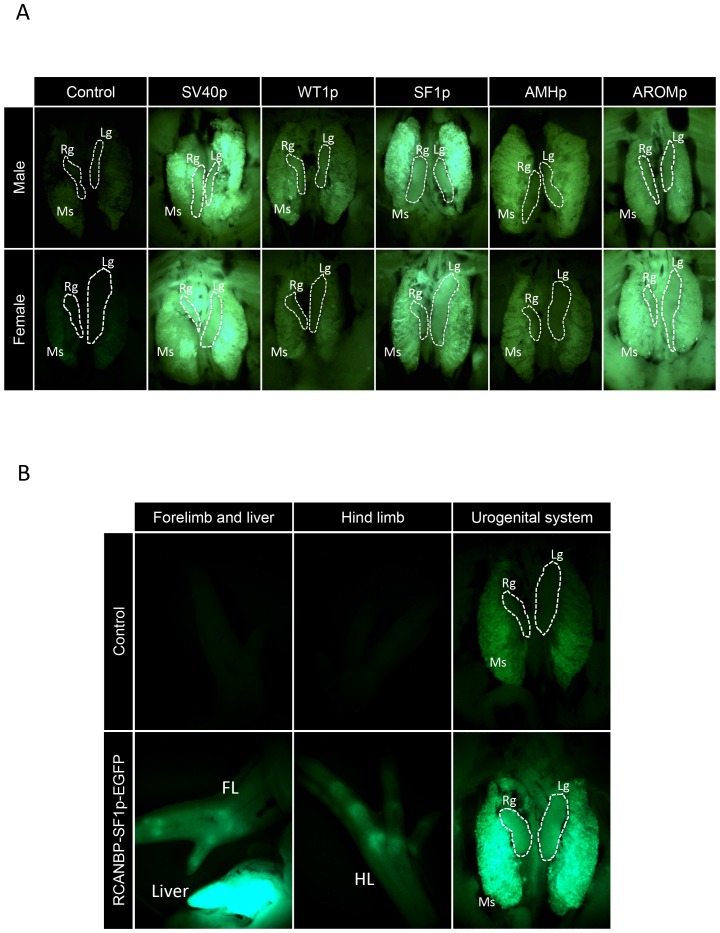
Wholemount fluorescent microscopy of novel gonad promoter expressed EGFP. Tissues from E7.5 embryos infected with RCANBP viruses containing *SV40* (*SV40p*), *WT1* (*WT1p*), *SF1* (*SF1p*), *AMH* (*AMHp*) and *aromatase* (*AROMp*) promoters. Dashed white lines delineate the left (Lg) and right (Rg) gonads, which sit on top of the mesonephros (Ms). A: Strong EGFP expression was evident for *SV40p* and *AROMp*, however, this was not confined to the urogenital systems. *WT1p* and *AMHp* produced low-level expression in the urogenital systems. EGFP expressed from *SF1p* was moderate in the urogenital system, and included the gonads. B: RCANBP-SF1p-EGFP infected E7.5 embryo; in addition to EGFP expression in the urogenital system, embryos also showed EGFP expression in the liver, forelimb (FL) and hind limb (HL).

To assess the ability of each of the promoter sequences to express a reporter gene in chicken embryos, RCANBP vectors were generated that contained each of the promoter sequences upstream of EGFP. High titre RCANBP viral stocks for each vector were generated and used to infect blastoderm stage embryos. The expression of EGFP was then monitored in E7.5 embryos by wholemount fluorescent microscopy. For each embryo, the expression of EGFP was first analysed for the whole embryo of both sexes. To test for gonad-restricted expression, the urogenital systems (mesonephros and gonads) were revealed by removing the viscera (Figure 2). Non-injected negative controls showed only background levels of fluorescence, whereas very strong EGFP was detected in the urogenital system for *SV40p* control in both sexes. In addition to the urogenital systems, EGFP expression in the *SV40p* infected embryos was evident throughout the entire embryo at high levels, indicating that as expected, this promoter exhibited ubiquitous transcriptional activity. For embryos infected with the RCANBP viruses encoding the various gonad factor promoters, a variety of EGFP expression patterns were observed. For *WT1p*, a low level of embryo-wide EGFP was evident that did not show any increase in the urogenital system. EGFP expression from *SF1p* was at low levels throughout the embryo, except for the liver and mesonephros, which both showed high levels of expression in both male and female (Figure 2B). For *AMHp*, EGFP expression was only evident in the urogenital system, as the rest of the embryo appeared to be negative. The level in the urogenital system however, was far lower than *SF1p*. The *aromatase* promoter produced strong EGFP expression throughout the entire embryo. The level of expression was very consistent across all tissues, including the urogenital system, and therefore did not show any tissue-specificity. Surprisingly, the mouse *SF1p* produced very weak expression overall, with no detectable expression in the gonads and a moderate level of expression in the liver (data not shown).

**Figure 3:**
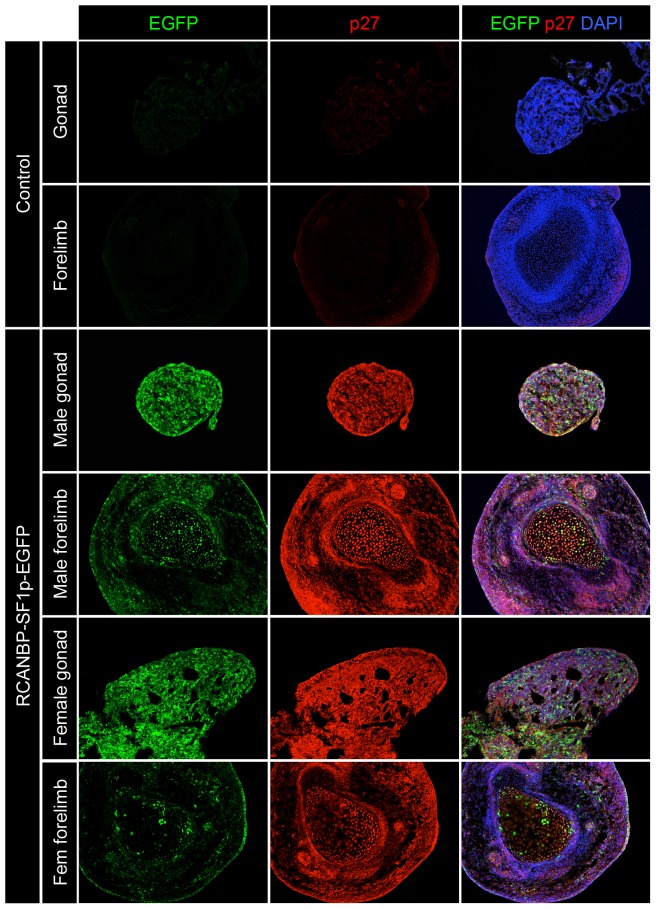
Tissue restricted EGFP expression from the *SF1* promoter. Immunostaining for EGFP (green) and the RCANBP viral antigen p27 (red), in RCANBP-SF1p-EGFP infected E7.5 embryos (magnification 10×). Control (male) gonad and forelimb tissues are negative for EGFP and p27. For both male and female embryos infected with the *SF1p* vector, EGFP expression is higher in gonad tissues compared to the forelimb.

Taken together, these data showed that each of the promoters tested produced varying activities in early stage chicken embryos. Since *AMHp* and *WT1p* showed only weak urogenital expression they were not pursued any further in this study. Although the *aromatase* promoter produced high-level EGFP expression, since it was not restricted to the urogenital system, it was also not pursued any further. The *SF1* promoter produced the most potentially useful expression pattern, as levels of EGFP were higher in the mesonephros and potentially the gonads compared to the rest of the embryo (Figure 2B). To further analyse the extent of its gonad-specific activity, the expression of EGFP was analysed by immunostaining. Embryos infected with RCANBP-SF1p-EGFP were dissected at E7.5 and gonad tissues were compared to samples of forelimb, which provided a representation of the rest of the embryo (Figure 3). Expression in the gonads of both sexes was evident, and although the forelimbs did show some immunoreactive EGFP expression, it was at greatly reduced levels compared to the gonads (Figure 3). P27 staining for the presence of viral epitope confirmed that the virus was present in each of the tissues.

**Figure 4:**
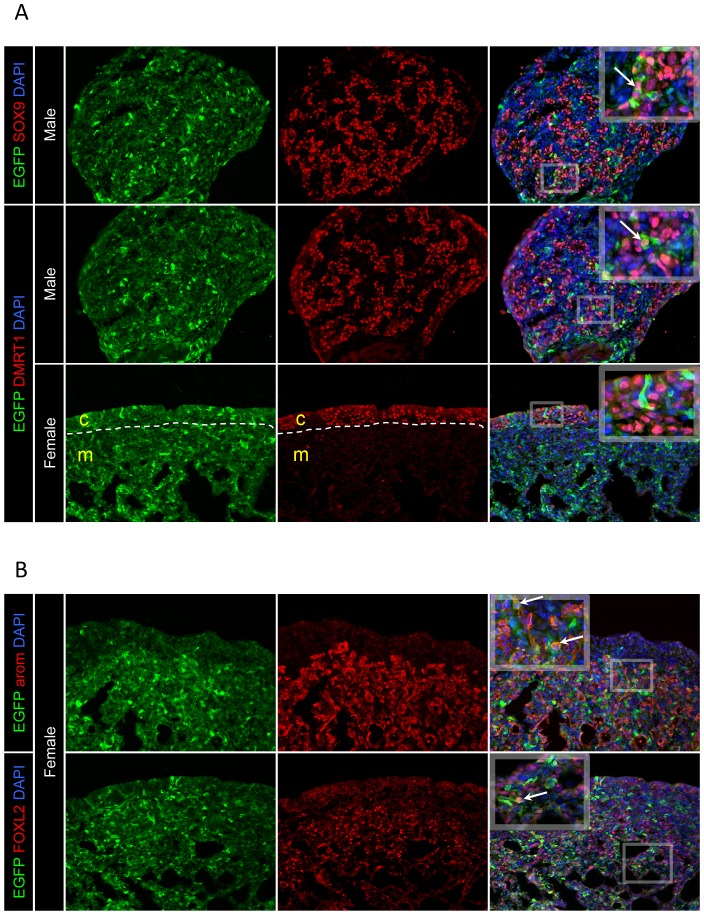
Cellular location of *SF1* promoter expressed EGFP compared to key gonad factors. Immunostaining for EGFP (green) and key testis and ovarian developmental proteins (red) in RCANBP-SF1p-EGFP infected E7.5 embryos (magnification 20×). A: Male genes: In male gonads, co-staining of EGFP with DMRT1 or SOX9 showed that both proteins were expressed in some cells simultaneously (white arrows). However, in female gonads DMRT1 expression did not overlap with EGFP. B: Female genes: In female gonads, co-staining of EGFP with aromatase or FOXL2 showed that both proteins were expressed in some cells simultaneously (white arrows).

The cellular location of EGFP expression from *SF1p* was then analysed in the context of other key sex development genes. The location of EGFP protein was compared to DMRT1, SOX9, aromatase and FOXL2 in the gonads of E7.5 embryos injected with RCANP-SF1p-EGFP. Analysis of the overlayed images of SOX9 and DMRT1 with EGFP revealed that in some cells EGFP was co-expressed (white arrows) with these proteins. Although, as EGFP is expressed in the cytoplasm and DMRT1 and SOX9 are expressed in the nucleus of cells in the cords of developing male gonads, these proteins did not co-localise (Figure 4). SOX9 is usually absent in developing female gonads, however, DMRT1 is expressed in female germ cells, which are located in the outer gonadal cortex at E7.5 (Figure 4A). The high power image of the cortex showed that although EGFP was expressed in some cells within the cortex, it did not appear to be expressed in any of the DMRT1 positive cells (i.e., germ cells, which are known to silence RCAS/RCAN viruses [12]).

Female pathway genes aromatase and FOXL2 were then analysed for localisation with *SF1p* expressed EGFP (Figure 4B). Aromatase showed strong cytoplasmic expression in the female medulla and closer analysis showed that EGFP co-localised with aromatase in some cells as indicated by the orange colouring (white arrows). In contrast, FOXL2 showed nuclear expression primarily in cells within the medulla, and when overlayed with EGFP, it was apparent that both of these proteins were in some cases present in the same cells (white arrows).

**Figure 5:**
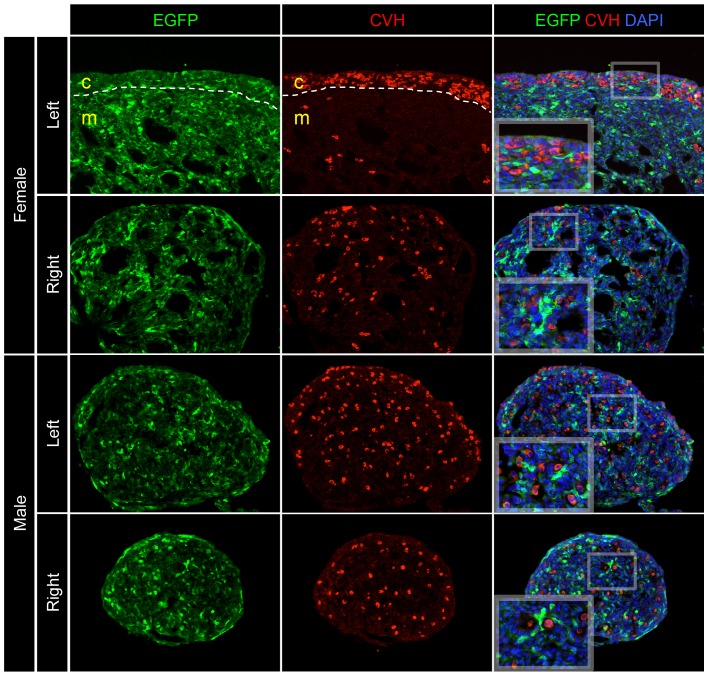
Cellular location of *SF1* promoter expressed EGFP compared to germ cells. Immunostaining for EGFP (green) and CVH (red) in RCANBP-SF1p-EGFP infected E7.5 embryos (magnification 20×). The expression of EGFP from *SF1p* did not overlap with any cells that were positive for the germ cell marker CVH.

To more closely analyse the relationship between EGFP and germ cells, the left and right gonads of both male and females were stained for the germ cell marker chicken vasa homologue (CVH). The left gonad of females characteristically shows predominant germ cell localisation within the thickened cortex, whereas the right gonad, and both the left and right gonads of males, show scattered germ cells localised throughout the medulla (Figure 5). When overlayed with EGFP, it was clear that in the left and right gonads of both sexes, germ cells in the cortex and medulla did not have any EGFP expression. Taken together with the DMRT1 staining in the female left gonad, these data suggest that *SF1p* does not express EGFP in germ cells when delivered from RCANBP. It does, however appear to show expression patterns that overlap with several key sex pathway genes in the somatic cells of both sexes.

### SF1p-mediated over-expression of DMRT1

**Figure 6:**
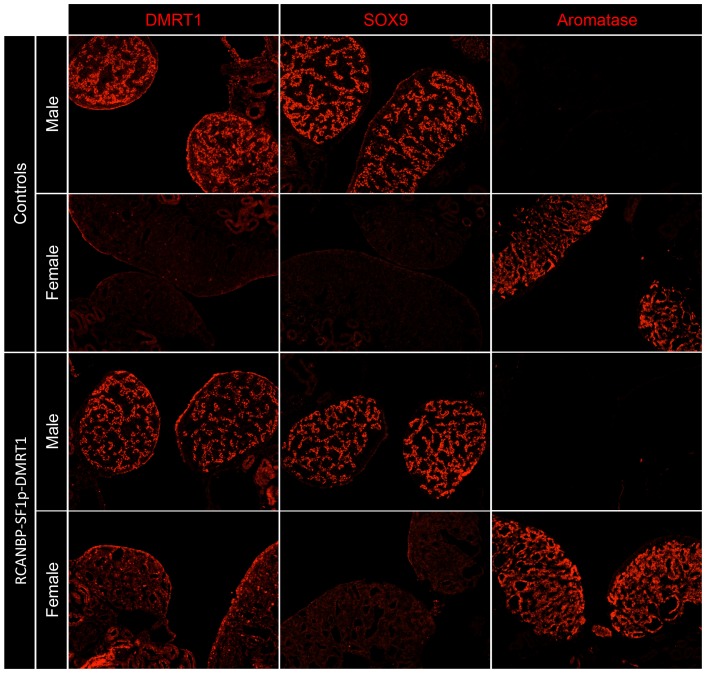
Over-expression of DMRT1 from *SF1p* in embryonic gonads. Immunostaining for key testis and ovarian developmental genes (red) in RCANBP-SF1p-EGFP infected E7.5 embryos (magnification 10×). Low-level over-expression of DMRT1 was evident in the gonads of RCANBP-SF1p-DMRT1 infected embryos compared to the control female. This over-expression did not cause any change in the expression of SOX9 in the female or aromatase in the male.

DMRT1 is normally expressed at higher levels in males compared to females and has been shown to be critical for testis development [9], [13]. We have previously attempted DMRT1 over-expression from RCASBP, which produces embryo-wide delivery via the viral LTR promoter, but found that it induced early stage embryo-lethality [13]. To test if *SF1p* could be used to over-express a testis pathway gene in female gonads, the *DMRT1* open reading frame was cloned downstream of this promoter sequence in RCANBP (called RCANBP-DMRT1). Embryos infected with RCANBP-DMRT1 at the blastoderm stage showed no signs of increased mortality or developmental abnormalities. Immunostaining showed that in female gonads DMRT1 was expressed at higher levels compared to the control female (Figure 6). However, this over-expression was not as high as that seen in the male control. In the RCANBP-DMRT1 infected male there did not appear to be any observable increase in the level of DMRT1 expression and none was observed outside the cords. However, we have previously seen that despite robust levels of RCASBP mediated over-expression of aromatase in both male and female gonads, no expression was observed in cell types that do not normally express this protein [23]. To analyse the effect of DMRT1 over-expression in female gonads, the expression of the male gene SOX9 and the female gene aromatase were analysed by immunostaining (Figure 6). Both the control and RCANBP-DMRT1 infected males showed robust SOX9 expression, while in the control female and in the RCANBP-DMRT1 infected female no SOX9 was detected. Strong aromatase expression in the control and the RCANBP-DMRT1 infected females was also evident, however, the RCANBP-DMRT1 infected male had no ectopic expression of this protein. These data indicated that despite increased levels of DMRT1 expression in female gonads, this was not sufficient to masculinize female gonads.

### SF1p-mediated over-expression of aromatase

**Figure 7:**
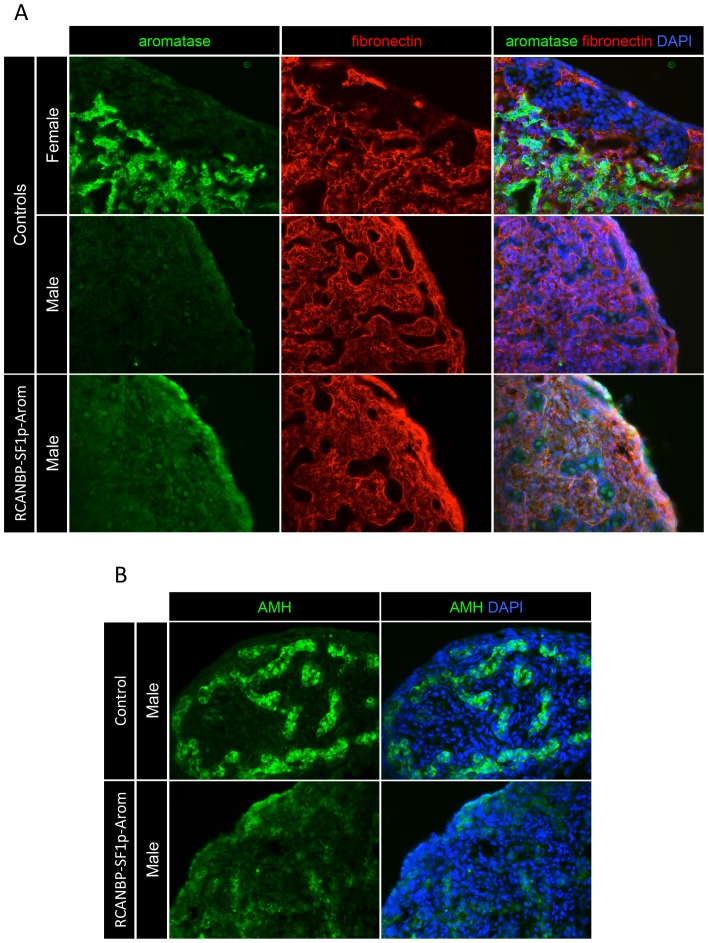
Over-expression of aromatase from *SF1p* in embryonic gonads. A: Immunostaining for aromatase (green), fibronectin (red) and DAPI (blue) in RCANBP-SF1p-EGFP infected E7.5 embryos (magnification 40×). Low-level aromatase over-expression was evident in the male left gonad compared to the control male. B: Immunostaining for key male protein AMH (green) and DAPI (blue) in RCANBP-SF1p-EGFP infected E7.5 embryos (magnification 40×). The over-expression of aromatase in the male gonad caused a reduction and disruption of AMH expression.

To test the ability of *SF1p* to over-express a gene involved in ovarian development in male gonads, the *aromatase* open reading frame was inserted downstream of *SF1p* in RCANBP (RCANBP-SF1p-Arom). Aromatase is normally expressed in a female-specific manner. We have previously reported that its global over-expression causes male-to-female gonadal sex reversal, which included the down-regulation of key testis genes and up-regulation of ovarian development genes [23]. The gonads of RCANBP-SF1p-Arom infected E7.5 embryos were analysed by immunostaining. The male left gonad had elevated aromatase expression compared to the control male, however this was much lower than the control female (Figure 7A). The control female had a characteristic thickened outer cortex and the male had defined cord structures and lacked a cortex region. Like the control male, the RCANBP-SF1p-Arom infected male also had cord structures and lacked a thickened outer cortex. The levels of the key male protein AMH were then analysed in RCANBP-Arom infected males (Figure 7B). Typical AMH expression was evident in the control male gonads, with strong staining throughout the cords. In contrast, in the RCANBP-Arom infected male AMH was reduced and its expression pattern was disrupted from its normal pattern. These data show that *SF1p* could be used to over-express a key female sex development factor in male embryos and was able to disrupt normal testis development.

## Discussion

In an effort to advance the methods available for studying sex development in avian species, we have characterized and compared several promoters for their activity in chicken embryonic gonads. Unlike other model systems such as the mouse, the production of transgenic animals for over-expression and knockdown studies is not yet routine for avian species. Therefore, the use of various techniques for the introduction of DNA vectors into live embryos is the most practical approach for mis-expression of genes.

Several gonad-specific expression systems have been described for the mouse, however these usually make use of large genomic fragments of genes involved in sex development to act as promoters for tissue specific expression. A major limitation on the use of the RCASBP and RCANBP for the delivery of recombinant sequences is a restriction on insert size of about 2.5 kb. Longer sequences may produce non-replication-competent viruses and thus lower titers [24]–[26]. The size of promoter sequences tested in this study was therefore limited to less than 1 kb and only the minimum predicted region required for activity was preferably used (which would then allow pairing an ORF of at least 1.5 kb in size). For *SF1p*, since the chicken minimal promoter was already validated *in vitro*, a fragment size of about 400 nt was used as this sequence was found to show similar activity to those tested up to about 1 kb [16]. Based on the analysis of the human *WT1* promoter [21], a fragment of about 600 nt was selected as this size provided the best expression. For *AMHp* a shorter sequence of about 300 nt was used, as it contained predicted binding elements that might confer activity.

Using these gonad sequences to transcribe EGFP *in ovo* from RCANBP provided the first indication of promoter activity. Although the expression of reporter genes from internal promoters in RCANBP has been previously reported *in vitro* [26] and in chicken embryos [14], activity in the gonads has not been described. Since strong EGFP expression from the viral LTR promoter can be seen in developing gonads from RCASBP [12], it was clear that this virus can effectively target this tissue. In the current study, infection with RCANBP containing the *SV40* promoter, embryos also showed strong EGFP expression throughout the entire embryo. Importantly, this included the urogenital system. This experiment provided a positive control and showed that expression from RCANBP in chicken gonads using an internal promoter was achievable.

It was anticipated that reporter expression driven from the various gonadal promoters should at least in part reflect some of the endogenous expression patterns of those genes. SFI is expressed endogenously in the gonads of both sexes prior to and during gonadal sex differentiation, but becomes female up-regulated as development proceeds [27]. Similarly, WT1 is expressed in the gonads of both sexes, but also in developing kidneys [27], [28], while AMH is only expressed in male embryonic gonads [29], and aromatase is entirely female-specific in embryonic gonads [30], [31]. However, the relevant core promoter fragments of these genes did not faithfully reflect the endogenous expression of these genes. EGFP reporter expression in E7.5 embryos varied from very low activity of the *AMHp* sequence to embryo-wide strong expression from the *AROMp* sequence. Endogenous aromatase is expressed female-specifically, so it was also interesting to note that the *AROMp* sequence provided strong embryo-wide EGFP expression in both sexes.

Taken together, these data therefore suggest that transcription of the native transcripts of these promoters requires additional up and/or downstream sequences or other structural features to achieve their normal patterns of expression. Taking the core promoter fragments out of context for testing in RCANBP clearly showed that most of these sequences could drive expression in non-gonadal sites, and thereby suggests that repressors or insulators were absent from the promoter regions used. Conversely, low-level reporter expression from promoters such as *AMHp* indicated that additional sequences are required for robust gonad-restricted expression. A relevant example of this requirement is demonstrated by a BAC transgene containing the mouse *SF1* gene 5′-flanking sequences. A 47 kb region can direct EGFP expression to the gonads, adrenal cortex, spleen and ventromedial hypothalamic nucleus in mice [32], but this fragment lacks important sequences that are present in a longer 111 kb version of the same region that can direct additional expression in the hypothalamus and pituitary [33].

The initial analysis suggested that *SF1p* showed the best potential for future application as a tissue-restricted promoter sequence. In particular, after the liver and mesonephros, the gonad showed the highest EGFP expression levels when using this promoter in RCANBP. There was however, low-level expression elsewhere in the embryo, particularly in the fore and hindlimbs (Figure 2B). Further analysis of *SF1p* expressed EGFP in gonads by immunostaining showed that compared to the forelimb (used to represent a non-gonadal tissue showing that showed some expression), the expression was higher in the gonads of both sexes compared to forelimbs. These data suggested that *SF1p* was a potentially an appropriate candidate for tissue restricted expression of sequences of interest.

Immunostaining gonads for EGFP expression along with various other gonad development genes provided a more in depth analysis of *SF1p* activity. Since EGFP was expressed in some of the same cells that were also expressing DMRT1, SOX9, FOXL2 and aromatase, *SF1p* could potentially be used to transcribe sequences in cell lineages that express key sex-determining genes. Staining for the germ cell marker CVH showed that EGFP was not expressed in the germ cells of neither male nor female gonads. This finding is consistent with previous observations that although RCAS-based viruses can infect germ cells, their transcriptional activity appears to be silenced, at least in embryos [12]. Therefore it does not necessarily reflect on the ability of *SF1p* to deliver expression in germ cells, as a non-RCAS vector might produce different results. It was also evident that EGFP expression from this promoter showed variegated expression in gonadal tissues (Figures 3, 4 and 5). The effect of viral integration site on transgene expression may at least in part account for this, especially considering that Rous Sarcoma Virus shows numerous insertion sites when infected either as a virus particle [34] or by DNA transfection [35], showing no apparent preference for specific integration sites.

A critical test for the potential use of *SF1p* for studies in sex development was to over-express genes involved in gonadal sex differentiation. To this end, the key testis development gene DMRT1 and the female-specific gene aromatase were tested. Previously, global DMRT1 knockdown in male embryos resulted in gonad feminization and ovarian development, which included the up-regulation of aromatase and down-regulation of SOX9 [13]. We have previously used RCASBP to globally over-express DMRT1 in chicken embryos, however infection with this virus induces early-stage embryo-lethality [13]. This was not surprising considering that it encodes a transcription factor regulating cell fate decisions [36], [37]. Recently, we reported that site-specific electroporation of RCASBP encoding DMRT1 into female gonads was able to avoid embryonic toxicity and activate testis pathway genes [9], however this method required a great deal of optimization, technical expertise and viral infection was limited to the left gonad. In the study reported here, embryos infected with RCANBP-DMRT1 showed normal survival rates, suggesting that embryo-wide expression was avoided. Given that DMRT1 over-expression was detected in female gonads (Figure 6), any non-gonadal expression was not at sufficient levels to induce embryo lethality at the time points analysed. Despite the elevated DMRT1 expression in female gonads, there did not appear to be any effect on the other sex development genes analysed. This was likely due to insufficient levels of DMRT1 being over-expressed (i.e. to match the level of a normal male), given that robust levels of DMRT1 over-expression in female gonads was required to induce male pathways genes, disrupt cortex formation and reduce aromatase expression [9]. It is therefore likely that a promoter stronger than *SF1p* would be required to match the level of expression required to effect gonad development. This notion is consistent with the hypothesis that a sufficient level of DMRT1 expression is required to initiate the testis developmental pathway [38].

Over-expression of aromatase in genetic male embryos from *SF1p* provided an example of the application of this sequence to over-express a female gene that is usually absent in male gonads. Aromatase over-expression in male embryos has been shown to override testis development and induce the ovarian program of gonad differentiation [23]. In the current study, it was found that despite only very modest aromatase over-expression in male embryos from *SF1p*, this was enough to disrupt the expression key male marker AMH. Although this phenotype was mild compared to that seen using the stronger viral LTR promoter of RCASBP [23], it did show that this promoter sequence can be used to over-express a gene to induce a gonad phenotype. The lack of effect of DMRT1 over-expression and positive effect of aromatase expression might be at least in part due to differences in structure and functions of these proteins. DMRT1 is a transcription factor that is expressed in the gonads of both sexes, and its level of expression most likely determines its activity in each of these tissues. In contrast, aromatase is an entirely female specific enzyme that can have a graded or transient effect when it is expressed at varying levels in male gonads, resulting in the formation ovotestis [23].

In summary, a number of potential gonadal promoters were tested for their ability to drive gene expression specifically in the embryonic urogenital system. *SF1p* offered a tissue-restricted expression pattern with high-level EGFP expression in the liver and mesonephros, moderate levels in the gonad, and lower levels elsewhere in the embryo. This sequence was capable of expressing EGFP in some of the same cells as key male and female gonad genes and was used to over-express the key female gene aromatase to induce a phenotype. It was also evident that this promoter would only be suitable for applications where a low or moderate level of over-expression is required. It will be interesting to see if SF1p can be employed for the expression of high efficacy short hairpin RNAs for RNA interference, especially since the use of weaker promoters *in vivo* are able to avoid cytotoxic effects seen with some stronger promoters [39]. In addition, there is the potential for linking *SF1p* with enhancer sequences, particularly with regions that might show gonad-specificity such as TESCO [40] and WT1 [21]. This study provides a novel method of achieving tissue restricted expression in embryonic chicken gonads and will be of use in the field of sex determination and gonadal development.

## Materials and Methods

### Ethics statement

All experiments were carried out with respect for the principles of laboratory animal care and were consistent with the *Australian Code of Practice for the Care and Use of Animals for Scientific Purposes, 7TH Edition 2004* and the *Prevention of Cruelty to Animals Act, Victoria 1986*. This included official approval from the Murdoch Childrens Research Institute Animal Ethics Committee (AEC # A627).

### Vector construction and virus preparation

Prior to insertion into RCANBP vectors, all promoters were first cloned upstream of EGFP in the transfer vector pSLAX. For the *SV40* promoter, the EGFP ORF was cloned from pEGFP-N1 (Clonetech) on *Nco*I and *Xba*I and ligated into pGL3-Promoter (Promega) digested with the same enzymes. This removed the luciferase ORF from pGL3-Promoter and relaced it with EGFP under the control of the SV40 promoter (*SV40p*). This vector was then digested with *Bgl*II and *Bam*HI and ligated into pSLAX digested with the same enzymes. For the other promoter sequences, a pSLAX vector encoding EGFP was first generated. The EGFP ORF was PCR amplified from pEGFP-N1 using a forward primer with an introduced *Hind*III site and a reverse primer with *Bam*HI, and then cloned into pSLAX on the same sites. All promoters were then amplified using forward primers with introduced *Eco*RI sites or *Nco*I sites and cloned into pSLAX with EGFP using the same enzymes. Primer sequences for *SF1p* were; forward 5′-ACCTCCCCGCAGTTCCTCTCTCCTGG-3′ and reverse 5′-AAGTACTCACCTCGATGCGGC-3′; *WT1* promoter: forward 5′-CATGCCATGGGAATTCATCTGCTCGCAGACCTTCTG-3′ and reverse 5′-CATGCCATGGGAATTCGCTAGCGAGCTCAGATCTGGGAGATATATTATACCACAGTAACCTC-3′; *AMH* promoter: forward 5′-TCACCATGGTCTAGATTCCACCCTCCTCTCCAA-3′ and reverse 5′-TCACCATGGTGTTCTGCTGCACCCACAG-3′, *aromatase* promoter: forward 5′-ATATAGAATTCGCGAGACCAAATCACAAGATAAA-3′ and reverse 5′- ATATAGAATTCGCAGGCTGGTGAAGTAGTTCAGTG-3′; mouse *SF1* promoter: forward 5′-TCACCATGGTCTAGACACACCCTTAGCCCAGCAGTC-3′ and reverse 5′-TCACCATGGTCCCAGGCCTCAGGTAGGGCA-3′. The promoter/EGFP cassettes were then excised from pSLAX using *Cla*I and ligated into RCANBP/A proviral DNA that had also been digested with *Cla*I. All constructs were verified by DNA sequencing. The viral DNA was then transfected into chicken fibroblastic DF1 cells using Lipofectamine 2000 (Invitrogen) and propagated for approximately 2 weeks. Recombinant virus was harvested from culture medium, concentrated by ultracentrifugation and titered as previously described [12].

### Embryo manipulation

High titer virus (approximately 10^8^ Infectious Units/mL) was injected into day 0 blastoderms, and eggs were sealed with parafilm and incubated at 37.5°C. Embryos were harvested on embryonic day 7.5 (HH32), as at this time point the first physical differences in male and female gonad development can be observed. These experiments involved at least 50 embryos for each experiment and at least 5 embryos per sex for each experimental condition were analysed. Immunostaining tissues with p27 antibody, which detects a viral epitope, confirmed RCANBP infection.

### Immunofluorescence

Tissues were fixed for 15 minutes in 4% PFA/PBS at room temperature, prior to processing for tissue section immunofluorescence, as described previously [41]. At least 5 embryos per time point and/or treatment were examined. Briefly, 10 µm sections were cut on a cryostat, permeablised in PBS 1% Triton X-100 and blocked in PBS 2% BSA for 1 hour. Primary antibodies were either raised in-house (rabbit anti-chicken aromatase (1∶5000), rabbit anti-chicken DMRT1 (1∶5000), rabbit anti-chicken vasa homologue (CVH) (1∶6000), rabbit anti-chicken FOXL2 (1∶6000), or were obtained commercially (rabbit anti-p27 (1∶1000); Charles River Services, goat anti-AMH (1∶1000); Millipore, rabbit anti-mouse SOX9 (1∶6000); Santa Cruz, mouse anti-fibronectin (1∶500); Serotec, goat anti-GFP (1∶500). Alexa-fluor secondary antibodies were used (donkey or goat anti-rabbit, mouse or goat 488 or 594; Molecular Probes). Sections were counterstained with DAPI.

### PCR sexing

Infected and control embryos were dissected at indicated time points. For genetic sexing of embryos, a small piece of limb tissue was digested in PCR compatible Proteinase K buffer and the genomic DNA was used for rapid PCR sexing [42]. By this method, only females show a W-linked (female-specific) *Xho*I band. Amplification of 18S rRNA in both sexes served as an internal control.
